# Institutional Care of Children in Low- and Middle-Income Settings: Challenging the Conventional Wisdom of Oliver Twist

**DOI:** 10.9745/GHSP-D-15-00228

**Published:** 2015-09-10

**Authors:** Paula Braitstein

**Affiliations:** ^a^​ University of Toronto, Dalla Lana School of Public Health, Toronto, ON, Canada

## Abstract

Whether institutions or extended families are better suited to care for orphans depends on the specific circumstances. Reported rates of traumatic experiences among orphans and vulnerable children are high in both institutions and extended families; improving the quality of care for such children should be the paramount priority in all settings.

See related article by Gray.

Acting in the best interest of the child is the fundamental principle that is supposed to guide local, national, and international policy, programming, research, and care for children. Most people would agree, yet there is a raging debate among scientists about how, what, where, and when to measure and interpret “best interests.” The stakes are high as there are millions of children lacking one or both parents (orphans) and millions more whose parents are alive but due to poverty or other circumstances such as substance abuse can’t, or at least don’t, provide them with an optimal care environment—or sometimes even one that meets their basic needs. Consequently, millions of children, especially in low- and middle-income countries, turn to the streets in an effort to take care of themselves.

On one side is a large body of evidence, mostly historical and from Eastern Europe and North America, about the negative physical and mental health outcomes of children in institutions.^1^ In essence, the evidence clearly demonstrates that socially and emotionally deprived environments are bad for children and that institutions caring for infants, particularly in Eastern Europe and North America, have embodied neglectful environments. As a result of this evidence, there are global demands for universal deinstitutionalization.

Yet the paper by Gray and colleagues published in *Global Health: Science and Practice* challenges stakeholders to perhaps rethink these sweeping conclusions.^2^ Their data, from a multicenter cohort of school-aged children in 5 low-income countries, indicate that children in family-based settings actually had a higher risk of physical or sexual abuse compared with those living in institutions, and children in family-based environments were as likely as those in institutions to report experiencing at least one potentially traumatic event. Although limited by the self-reported nature of the data, these findings are supported by a rapidly growing body of evidence to suggest that, at least in low- and middle-income countries, institutional environments may sometimes be better equipped and prepared to care for children in need than many extended families and other family-based configurations of care.^3^^-^^8^

It is a counter-intuitive idea. Charles Dickens brought us *Oliver Twist* and with it enduring social and cultural icons of institutions being inherently harmful for children. Residential schools in Australia, Canada, and the United States have strongly reinforced this idea by leaving aboriginal communities and First Nations struggling to cope with lasting multigenerational trauma resulting from the schools. When Nicolae Ceauşescu was eventually deposed and Romania exposed to the outside world, horrifying images of children in institutions imprinted themselves onto our collective mind with the undisputed message that institutions are bad for children.

Truly, it is doubtful that any child would wish *a priori* to grow up in an institution, and there is no denying the potential for neglect and severe abuse to occur in institutions in the absence of appropriate resources, regulation, and oversight. The question is whether institutional environments themselves are *necessarily* bad environments for children. The vast majority of data about children in institutional environments has come from high- and very high-income settings, and often without comparison groups. This literature may be subject to publication bias, because the more shockingly negative findings are, the more likely they are to get published. The horrific images and data from children in Romania stay with us and lead us to draw the strong conclusions to which Gray and her coauthors allude: institutions should all be closed. But steadily, piece by piece, there is another picture emerging that just maybe, given the widespread poverty, rapid urbanization,^9^ and high dependency ratios that characterize many extended families in sub-Saharan Africa^10^ and Asia, institutions are not so bad for children in those places, particularly when compared with their compatriots living with extended family. At least, they appear to be no worse.

A growing body of evidence suggests institutions may sometimes be better equipped to care for children in need than extended families, especially in low- and middle-income countries.

Regardless of which side of the deinstitutionalization debate one takes, what also especially deserves attention from this paper is the fact that over 90% of participating children in *both* environments reported experiencing at least one potentially traumatic event *other than* the death of a parent.^2^ Over 50% of both groups reported at least one episode of physical or sexual abuse by age 13, and physical or sexual abuse was the leading cause of incident trauma in the cohort. One might surmise these to be unsurprising findings for children living in institutional environments given the background circumstances that presumably led to them becoming institutionalized. However, the fact that so many children in both care environments experienced potentially traumatic events—and physical and/or sexual abuse in particular—is alarming. These rates are unacceptably high, but why could they be higher in families than in institutions?

There are several theories, all of which need testing. One is that at least in some settings, the majority of institutions are funded through adequately resourced religious organizations and so are better able to respond to the material needs of children.^5^ There is evidence that orphaned girls, especially, exchange sex for material goods in order to survive; if this need is taken away their risk of sexual abuse will be lessened.^11^^,^^12^ It may also be possible that many of the staff, or at least founders, of institutions in low-income settings are working in, or have set up, the institution explicitly because they want to take care of children. In contrast, there is a broad literature indicating many extended families are forced into caring for orphans because of family or cultural expectations. They may already be struggling financially and view the orphaned child as an added burden. There is a body of evidence that indicates that orphans are often systematically discriminated against within the household in which they are living.^13^^,^^14^ In essence, extended families in many areas are stretched to the limit financially and perhaps emotionally. Families are not generally supported to care for orphaned children although thanks to support from governmental and nongovernment organizations, more and more families are receiving small cash subsidies. The subsidies do seem to help significantly.^15^

Extended families in low- and middle-income countries may be financially, and perhaps emotionally, stretched to care properly for orphans.

The evidence is mounting that at least in some places, institutional environments actually create family-like environments.^5^ Moreover, there is plenty of evidence including the paper by Gray et al. that illustrates a high burden of abuse and neglect among orphans living with family.^16^ Indeed the one potentially traumatic event that was higher for institutionalized children in Gray et al.’s study was being forced to leave the home or care setting—the authors speculate that this is likely due to the closing of institutions in which they were living.

Thus, the answer to the question posed earlier appears to be: “No, there is nothing *necessary* about living in an institution that seems to be inherently bad for a child.” As more and higher quality data from low- and middle-income countries emerge, the idea that it is the quality and characteristics of the care within an environment, rather than the type of environment itself, that most likely impacts a child’s health and well-being, is likely to grow.

In many ways, the debate on institutionalization is asking the wrong question. The questions we should be asking ourselves are how do we prevent traumatic events among vulnerable children across the board—regardless of the care environment? How do we create a world in which most children can grow into their teens without experiencing a potentially traumatic event? How do we support those who have experienced a traumatic event? How do we support families to take better care of the children in their charge in the context of poverty, culture, urbanization, and the rapidly changing world we are all trying to adapt to? What is the role of institutions for children in need of a safety net? How should they be structured to respond adequately to the physical and emotional needs of infants, toddlers, young children, and adolescents? What are the optimal care characteristics within any environment that maximize resiliency and break the cycles of poverty, exploitation, and abuse among young people?

The quality and characteristics of care within an environment might be more important to a child’s health and well-being than the type of environment itself.

These are the next questions that need to be asked—and answered—by scientists engaged in the debate about what is in the best interests of vulnerable children globally. One can only hope that all stakeholders in the discussion can keep open minds, let the evidence evolve, and develop best practices that are truly in the best interests of children in all regions of the world—ones that do not hang on the image of poor little Oliver Twist.
